# Cleavages and co-operation in the UK alcohol industry: A qualitative study

**DOI:** 10.1186/1471-2458-12-483

**Published:** 2012-06-26

**Authors:** Chris Holden, Benjamin Hawkins, Jim McCambridge

**Affiliations:** 1 Department of Social Policy & Social Work, University of York, Heslington, York, YO10 5DD, UK; 2 London School of Hygiene and Tropical Medicine, 15-17 Tavistock Place, London, WC1H 9SH, UK

**Keywords:** Alcohol, Industry, Policy influence, Lobbying, Minimum pricing

## Abstract

**Background:**

It is widely believed that corporate actors exert substantial influence on the making of public health policy, including in the alcohol field. However, the industry is far from being monolithic, comprising a range of producers and retailers with varying and diverse interests. With a focus on contemporary debates concerning the minimum pricing of alcohol in the UK, this study examined the differing interests of actors within the alcohol industry, the cleavages which emerged between them on this issue and how this impacted on their ability to organise themselves collectively to influence the policy process. We conducted 35 semi-structured interviews between June and November 2010 with respondents from all sectors of the industry as well as a range of non-industry actors who had knowledge of the alcohol policy process, including former Ministers, Members of the UK Parliament and the Scottish Parliament, civil servants, members of civil society organisations and professionals.

**Methods:**

The paper draws on an analysis of publicly available documents and 35 semi-structured interviews with respondents from the alcohol industry (on- and off-trade including retailers, producers of wines, spirits and beers and trade associations) and a range of non-industry actors with knowledge of the alcohol policy process (including former Ministers, Members of Parliament and of the Scottish Parliament, civil servants, members of civil society organisations and professional groups). Interviews were recorded, transcribed and analysed using Nvivo qualitative analysis software. Processes of triangulation between data sources and different types of respondent sought to ensure we gained as accurate a picture as possible of industry participation in the policy process.

**Results:**

Divergences of interest were evident between producers and retailers and within the retail sector between the on and off trade. Divisions within the alcohol industry, however, existed not only between these sectors, but within them. Cleavages were evident within the producer sector between different product categories and within the retail sector between different types of off-trade retailers. However, trade associations were particularly important in providing a means by which the entire industry, or broad sectors within it, could speak with a single voice, despite the limitations on this. There was also evidence of ad-hoc cooperation on specific issues, which resulted from both formal and informal contacts between industry actors.

**Conclusions:**

Alcohol industry corporations and trade associations collaborate with one another effectively where there are shared interests, allowing the best placed bodies to lead on a given issue. Thus, whilst industry actors may be deeply divided on certain issues they are able to coordinate their positions on occasions where there are clear advantages in so doing. Health policymakers may benefit from an awareness of the multiplicity of interests within the industry and the ways that these may shape collective lobbying positions.

## Background

Over the past four decades there has been a sharp increase in the affordability of alcohol in the United Kingdom (UK) [1]. This fall in the price of alcohol in relation to income has coincided with an increase in the aggregate levels of alcohol consumption and alcohol related harm in the UK [2,3]. Furthermore, the trend during this period has been for a shift in sales from the on-trade to the off-trade, which has resulted at least in part from the expansion of national supermarket chains and their use of discounted alcohol as a key component of their promotional activity [4]. This combination of factors has resulted in policy debates – initially in Scotland and subsequently in Westminster – gravitating towards price as a mechanism for reducing rates of alcohol related harm. In 2009, the Scottish Government proposed legislation which introduced a series of measures designed to reduce the availability of alcohol, including a minimum price of 45p per unit [5]. The Alcohol Etc (Scotland) Bill was passed on 11 November 2010 but, following widespread opposition from sections of the alcohol industry and opposition MSPs, the clause introducing minimum unit pricing (MUP) was removed. The new SNP majority administration elected in May 2011 subsequently introduced a further Bill to the Scottish Parliament to implement MUP. The Conservative-Liberal Democrat administration in Westminster announced similar plans to legislate for MUP in March 2012 [6]. The present study focuses on the debates surrounding pricing and promotions policy in England and Scotland between May 2007 and November 2010.

The alcohol industry has generally been seen as opposed to public health interventions based upon price. Furthermore, a number of commentators have made reference to the perceived influence of the industry on government policy and have called for more research on its activities [7-10]. However, despite a growing literature on the role of corporate actors in other areas of public health policy-making, such as tobacco [11], there have been very few studies of the role of industry actors in the alcohol policy process [12-14]. Furthermore, in the calls for more research, there has been little consideration of exactly what constitutes ‘the industry’. As Jernigan [15] points out, the alcohol industry is composed not just of producers of different kinds of beverages but also of distributors and retailers, including those engaged in the sale of alcohol through both the on-trade (pubs, bars, restaurants and nightclubs) and the off-trade (supermarkets, convenience stores and off-licences). Whilst the diverse range of actors within the industry has been commented upon before [16], this is the first study to examine how the dynamics between these players affect their ability to organise politically. We discuss the specific lobbying strategies of alcohol industry actors elsewhere. This article examines the differing interests of the range of actors comprising the alcohol industry, the cleavages which emerged between them on the issue of government intervention on alcohol pricing and how this impacted on their ability to organise themselves collectively in order to influence the policy process.

## Methods

Investigating internal industry or corporate processes is inherently difficult due to problems of access and the risk that, where access is obtained, this may not result in meaningful data. This is particularly the case where corporate influence on the policy process is being investigated, since corporate actors may be reluctant to share information that is not already publicly available. Research on the activities of tobacco companies in influencing the policy process has relied overwhelmingly on internal industry documentation released as a result of litigation [17]. Nevertheless, semi-structured interviews have been utilised successfully in a number of studies of corporate political influence, such as that by Braithwaite and Drahos [18]. Despite the limitations of such interviews, they are the most feasible means of gaining insight into corporate actors’ political strategies in the absence of access to internal documents.

The present study began with a preliminary stakeholder analysis [19,20] based on a review of relevant literature, documents and our existing knowledge of the alcohol industry and policy process. This provided the basis for purposive sampling of potential respondents who were approached for semi-structured interviews via email, telephone or in person. We also made use of snowball sampling, whereby initial respondents were asked to help identify other relevant informants. All potential respondents were provided with written details about the nature and aims of the research before deciding whether to participate. This highlighted the focus of the project on alcohol policy making in the area of pricing and promotions policy and the role of corporate actors within it. All respondents were asked to sign a consent form confirming if the interview could be recorded and indicating their willingness to be quoted and/or identified in any subsequent outputs. A number of respondents agreed to be interviewed on condition of anonymity and/or confidentiality. Ethical approval was obtained from the London School of Hygiene and Tropical Medicine research ethics committee.

A range of actors engaged in the alcohol policy-making process at both Westminster and Edinburgh were identified and interviewed. Whilst not all those approached agreed to participate, respondents came from all sectors of the alcohol industry, including the on and off trade; producers of wines, spirits and beers; retailers of various kinds; and involving both individual corporations and trade associations. Our sample also included a range of non-industry actors who had knowledge of the alcohol policy process and the involvement of corporate actors, including former Ministers, Members of Parliament and of the Scottish Parliament, civil servants, members of civil society organisations and professional groups. Interviews with these respondents were then triangulated with those with industry respondents in order to build as accurate a picture as possible of industry participation in the policy process. These interviews were also informed by, and triangulated with, the public statements of the various industry actors on MUP, particularly in response to consultative exercises undertaken by the Westminster and Edinburgh governments. A total of 35 interviews were conducted between June and November 2010. Table 1 sets out the number of respondents from each sector and indicates the number of potential respondents approached who did not respond to our request for an interview, or did not wish to participate in the study.

**Table 1:** Breakdown of interview respondents by sector

**Sector**	**Interviews Conducted**	**Did not Respond**	**Declined Interview**	**Total**
Producer companies	5	2	4	11
On-trade operators	2	2	0	4
Off-trade retailers	2	2	1	5
Industry Trade Associations	13	0	0	13
Government	7	29	12	48
Public Health	6	0	1	7
Total	35	35	18	88

With respondents’ permission, interviews were recorded and the transcripts of these analysed using Nvivo software. Whilst a number of themes emerged from the interviews, the analysis presented here focused upon intra-industry dynamics and their implications for the making of alcohol policy. Major themes which emerged from the project include the various political strategies of industry actors, the industry ‘framing’ of key alcohol policy issues and the impact of devolution in Scotland upon alcohol policy [21]. Analysis for this article is based upon identification of the key cleavages within the industry, as revealed by the interview data and its triangulation with relevant publicly available documents. Furthermore, our analysis is restricted to the issue of pricing and promotions policy and related issues and does not investigate the different cleavages which exist between industry actors on other issues. In order to improve the reliability of the analysis, both the researcher who had conducted the interviews (BH) and the first author (CH) listened to recordings of them before agreeing initial key themes, allowing for an iterative process of refinement as the final analysis was undertaken.

### Findings

The alcohol industry is composed of companies with often widely differing strategic interests and organisational cultures. One representative from a national trade association commented that:

"This is a very, very fragmented industry. No one likes each other very much; the on-trade hates the off-trade, wine and spirit and beer companies they’re all arguing about various things."

Similarly, a representative from a national chain of supermarkets argued that ‘…the only thing that everybody has in common is that … they want to be able to continue to sell alcohol.’

In order to understand the dynamics of the alcohol policy debate in the UK it is necessary therefore to examine the positions adopted by different industry actors on specific issues and the degree to which they were able to form alliances and act collectively. The coalitions of interests which exist between different actors lead to the emergence of a number of cleavages between different companies and sectors of the industry. There are cleavages evident between producers and retailers and, within the retail sector, between the on-trade and the off-trade. The divisions within the alcohol industry, however, exist not only between sectors, but within them. Further cleavages are evident within the producer sector between different product categories and within the retail sector between the different types of off-trade retailers. We examine each of these cleavages in turn before discussing the role of trade associations in managing the tensions between their member companies and their links with associations in different sectors of the industry. Finally, we examine rationale behind actors’ attempts to co-ordinate their public affairs activities.

### Producers and retailers

The increasing focus on the retail arm of the alcohol industry in debates about alcohol related harm has led to the emergence of a cleavage between retailers of alcohol on the one hand and producers on the other. In addition, certain producers claimed to be dissatisfied with the way in which their products are sold by certain supermarkets. There appeared to be a tension between the desire of producers to market their products as premium brands and the desire of supermarkets to use discounted alcohol to attract customers into their stores and so increase sales. As a representative of a producer organisation commented:

"I think the reason why Molson Coors will advocate minimum pricing is that they find it very difficult to build their brands through supermarkets because supermarkets keep on discounting them, they keep on selling them cheaply, they’ll sell them in conjunction with other brands so it will be buy any two out of Carling, Carlsberg, Fosters, which creates in the consumer’s mind this idea that all the brands are interchangeable and it undermines all causes and attempts to build up a certain profile for their brand, to build up a certain image for it etc. The supermarkets will just reduce it all to: It’s just liquid. It’s just liquid to get down your neck, kind of thing."

This view was echoed by MSP Michael Matheson who, as a member of the Scottish Parliament’s Health and Sport Committee, was involved with the development of the Scottish Government’s proposed legislation introducing MUP:

"And because they have premium brands, they aren’t really happy with the way in which the supermarkets are squeezing them on their margins, because supermarkets are saying well we want to stock high and sell cheap, and if you want your products in our place you’re going to have to play that game I’m afraid, guys."

The supermarkets’ strategy had a number of consequences for the business models adopted by brewers. The downward pressure exerted on profit margins meant profits could only be achieved through a higher volume of sales. This ran counter to the strategy of some brewers, such as Molson Coors, whose business model was based around lower production volumes of higher margin, premium products. A representative of the company explained that this was behind their decision to support discussions about the possibility of banning below cost selling:

"Molson Coors is open around below cost selling and minimum pricing – none of our competitors are. We took a conscious decision about eighteen months ago to increase our prices in the market, and to cut volume, and that has helped us… we think that’s part of actually changing beer… the beer agenda. Since then some of our competitors have followed us on that, and others haven’t; so very different strategies and beliefs."

The tensions between the producers and the off-trade are not limited to the frustrations felt by certain producers about the business practices of the supermarkets. Representatives of the off-trade were also critical of how they thought producers had stymied the industry’s efforts to promote responsible drinking:

"I’m cynical, listening to some of the stuff about producers and I remember the battle that we had originally with Department of Health, the producers did with their original health label. Our members looked at it and thought, “Okay. Looks okay. It’s a health label. All it’s doing is telling people this has got 2 units, don’t drink more than 4.” The producers were completely against that because they saw it as the next step towards the tobacco smoking ban: the big pictures with labels on it."

### The on-trade and the off-trade

In addition to the cleavages which exist between producers and retailers, there are divisions evident between different branches of the retail sector. These are principally issues in which the interests of the off-trade are set against those of the on-trade. Representatives of the on-trade felt that they had been unfairly treated by recent legislation in comparison to supermarkets and that this was leading to a decline in on-sale revenues. As a respondent from a pub operator commented:

"I think that legislation for the on-trade is absolutely sensible and so there’s no happy hours and all that sort of stuff. So that’s fine, no irresponsible promotions. What there is though is a strange dichotomy, a difference between the on-trade and the off-trade. The off-trade, you can buy whatever, 18 pints for a tenner. You can drown in that. But there’s different rules for the on-trade and the off-trade."

Other respondents, such as the Secretary of the All Party Parliamentary Beer Group, felt that pubs were the victims of a concerted campaign by supermarkets to drive people from the on-trade to the off-trade:

"It is a cut-throat business and it appears pretty clear that the major players have been quite deliberately pursuing a strategy which… drove the pub out of business in order to add further impetus to the shift from the on to the off-trade. But they’ve done it before. They did it with greengrocers and they did it with butchers, they did it with bakers. That’s what they do – they drive other people out of business. Why shouldn’t they do it with pubs?"

Some respondents from the off-trade resented this view, with one representative of a retailers’ trade association referring to a ‘real victim complex that’s been developed in the pub trade over the last four or five years, partly down to the sheer weight of closures of pubs.’ Supermarkets operators, however, sensed a shift in the climate of the debate around alcohol policy, with some expressing the view that their businesses had become an ‘easy target’ for both legislators and the media.

### Intra-sectoral cleavages

It is perhaps not surprising that there are differences of opinion or conflicting interests between different sectors of the industry. However, cleavages between actors are evident within sectors of the industry as well as between them. Within the producer sector the differing interests of beer, wine, spirit and cider producers play off against one another. Within the retail sector, the interests of the off-trade are distinct from those of the on trade. Within these subsectors too there are differences between large-scale multiple retailers, such as supermarkets and pub companies (pubcos) and smaller, often independent, retailers and publicans.

#### Producers

Within the producer sector there are a number of cleavages evident between different product sub-sectors. Principally, these disputes centre on the differential levels of tax and duty levied on different types of products such as beer, cider and spirits. The first of these cleavages is between beer and spirits producers. Whilst spirits producers have argued for equivalency in the rates of duty levied on alcoholic drinks – which would create a direct relationship between the volume of alcohol within a product and the level of duty levied upon it – beer producers reject this idea since it would lead to a sharp increase in the price of beer relative to spirits. They argue instead that the lower levels of duty levied on beer should be maintained on the grounds that it is a low alcohol product and thus should be promoted as a healthier choice for consumers than stronger alternatives.

A further cleavage emerged between the beer producers and cider producers, again over the issue of taxation. Whilst beer producers argued that beer and cider are equivalent products which should therefore be treated similarly by the taxation system, cider producers were keen to highlight the particular costs involved in cider production – and the economics of apple production – which necessitated government support for the industry through the tax system.

The main policy difference between cider and spirits producers focussed not on the issue of taxation but on the debates surrounding underage drinking, binge drinking and related public order issues. Representatives of the cider industry were keen to argue that it was not their product which was responsible for these issues and sought to lay the blame instead on cheap spirits and spirit based ‘alcopops.’ Spirits producers, meanwhile, claimed the exact opposite: that cheap ciders and strong lagers, rather than spirits, were the issue.

In addition, divisions emerged between producers within the same product category. This may occur because the brand portfolios of certain companies traverse different drinks categories, requiring them to adopt nuanced and carefully formulated positions on issues affecting one sector of the market in such a way that it would not affect its interests in another. Similarly, these positions may reflect very different marketing strategies and perhaps even corporate cultures between different brewers. Differences of opinion between producers were most evident in the beer sector with some producers, such as Tennent’s and Molson Coors, coming out in favour of MUP in Scotland and others strongly opposing the measure. As the representative of Molson Coors commented:

"in this industry you have very conflicting opinions, you have very different market practices. So you’ll have some companies who are… selling it at bonkers prices […] So you have very conflicting, almost industry destroying strategies from some competitors who think this is an OK way to behave, and then you have other people that try and take a more balanced, nuanced view. You have people that are anti-regulation; so you have some kind of real tensions."

Producers have thus found it difficult to adopt a clear and coherent position on pricing policy or to present a consistent message to government on this issue.

#### The off-trade

Within the off-trade sector, there are a number of cleavages evident which reflect the differing interests of particular companies. The off-trade includes an incredibly diverse array of retailers in terms of scale, ranging from the largest supermarket chains down to independent convenience stores. As a representative of the Association of Convenience Stores (ACS) highlighted, the issue of below cost selling is an area in which the interests of small and independent retailers diverge from those of larger chains of supermarkets which are able to use discounted alcohol to attract customers into their stores:

"The issue that we’re more interested in, even more than we are in alcohol, is the issue of the power of supermarkets; and… we believe that supermarkets … should be prohibited from selling low-cost across the whole product range of what they do, because we think that is… that has predatory impacts on competition in the broader marketplace."

Consequently, the ACS were open to the possibility of a ban on discounted sales of alcohol and would seek to try to influence the details of any prospective pricing intervention in such a way as to benefit the interests of their membership.

In addition to the cleavages which exist between supermarkets and smaller retailers, there are differences in business strategy and policy position which exist between the largest supermarkets operators. For example, whilst Tesco and Morrisons tentatively welcomed the possibility of a ban on below cost selling, others were more reticent about the desirability or the effectiveness of this type of policy. A further point of contention centred on how the cost of alcoholic beverages would be calculated in any policy which sought to prohibit below cost selling. The representative of one national supermarket chain was concerned about the possibility of this being based on invoice price: the price at which each supermarket purchases alcohol from producers. This, they feared, would hand a competitive advantage to Tesco, the largest UK retailer, which due to its size may be able to negotiate lower purchase prices with manufacturers than other supermarkets.

Some representatives from other supermarkets felt that the decision by Tesco to support a ban on below cost selling was motivated by the competitive advantage it would hand them in relation to other supermarkets. As the current market leader, a ban on discounting could consolidate Tesco’s position within the alcohol market. Furthermore, it would prevent its competitors using alcohol pricing as a means of gaining wider market share from Tesco.

### T**rade associations**

The crucial role played by trade associations in the public affairs work of both alcohol producers and retailers is reflected in the number of associations active within the alcohol industry. In the producer sector in particular, companies are often members of multiple associations which focus on a particular aspect of their business or which represent their interests in a specific forum or at a particular level of government. Within the field of alcohol policy, the main trade associations are the British Beer and Pub Association (BBPA) and the Wine and Spirits Trade Association (WSTA). The interests of other groups are represented through the National Association of Cider Makers (NACM), the Gin and Vodka Association (GVA), the Association of Licensed Multiple Retailers (ALMR), which represents the interests of larger pub operators, and NOCTIS, representing nightclubs and the late night sector. In terms of the off-trade, the British Retail Consortium (BRC) represents the interests of supermarkets and other retailers whilst the Association of Convenience Stores (ACS) represents smaller scale retailers.

The BBPA represents not only the interests of brewers and marketers of beer, but those of publicans and the pub industry. Similarly, the remit of the WSTA is to represent the interests of wine and spirits producers and importers as well as off-trade retailers. This division of labour reflects a coalition of interests between particular producers and routes to market. The WSTA’s concern with the off-trade is a consequence of the fact that the majority of wine and spirits in the UK are sold through the off-trade. Similarly, whilst increasing volumes of beer are now sold through supermarkets and other off-trade retailers, the alliance between brewers and on-trade retailers evident in the BBPA was forged from the historical connections between the brewers and the pubs through which their beer is sold.

Trade associations representing a single product category, such as the NACM, or a single section of the industry such as the ALMR, felt they were able to adopt a united position on most issues of importance to their membership. However, given that the larger trade associations such as the BBPA, the WSTA and the BRC are broad churches – containing members from different sectors of the industry and with varying interests – it is often difficult for them to speak with a single, uniform voice on a given issue. The potentially conflicting interests of different groups must be taken into account in order to arrive at positions on which most members can agree.

Consequently, the arguments made by trade associations often represent the lowest common denominator on which all members can agree and may affect the ability of the association to articulate the views of its members to government. The BBPA, for example, faced problems in arriving at a common position on pricing policy given the differing perspectives of pub owners and producers and the differences of opinion which existed between producer members on the issue. Given the differing views on these issues, a number of BBPA members felt that the association was perhaps not as effective a channel for engaging government as it could be. As the representative of a brewing organisation commented, the diverse membership of the BBPA makes it difficult for the organisation to articulate the views of its members clearly and effectively:

"We are letting BBPA to be the ones that will go to the government and express our views. What makes it a little bit complicated, the BBPA is the beer and pub association. Actually, the big brewers are all members but then you have all the pub companies and the local brewers, most of them. We don’t always have the same view."

The problems faced by trade associations in agreeing on a common position on the most commercially sensitive issues was highlighted by the decision of Tesco, the UK’s largest retail operator, to support some form of government intervention on alcohol pricing. This ran counter to the agreed policy position of trade associations such as the BRC and the WSTA of which it is a member. Whilst elements of dissent are inevitable within any trade association, a departure from the agreed line by the biggest and potentially most influential member poses clear problems. As a representative from one such association commented:

"Without a doubt it’s made our job harder, yeah. But they were kind enough to tell us they were going to do it, and talked to us about it, and allowed us the opportunity to discuss and understand why they wanted to do it, and I think we’re fairly clear on what’s behind the decision etcetera. It does make life much more difficult, but a conversation was had which went along the lines of ‘the rest of the membership doesn’t want to change their position on minimum pricing’, that’s fine, we accept that. They didn’t… and at no point have they asked us to change our position."

Whilst certain organisations such as Tesco could break ranks with the agreed positions of the trade associations which represent them, this option was not available to all companies whose size, political influence and resources meant they had little choice other than to rely on trade associations to represent their views. Great importance was placed by these companies on ensuring that the line agreed by the association took into account their particular perspective on a given issue. As the representative of one retail company commented:

"Yeah, I mean trade associations you can throw money at them, but unless you participate and you develop the relationships within the trade association your voice isn’t going to be heard. […] I mean I spent, you know, like yesterday, I spent an hour on the phone talking to the food policy lead at the BRC, talking about his first draft of the licensing submission. Now if I hadn’t done that; if I hadn’t talked to him for an hour, and I hadn’t done it at that stage, he would have written something quite different."

Despite the obvious challenges faced by trade associations and the frequent difficulty they experienced in arriving at common positions, almost all respondents felt that they remained important actors in the policy process. By representing the industry – or a particular sector of it – collectively, trade associations were often able to influence policy debates more effectively than companies acting unilaterally. This view was shared even by those respondents who had been critical of certain aspects of their trade association’s work:

"We go through the collective organisations because we also believe […] we would like to have one view as an industry because this is the better way to do it and this is also why associations are there, to represent us. And even if we don’t agree with everything that others say, it’s better to have a common view than to come up as a split kind of tribe."

Whilst the largest companies recognised the value of trade associations, their ability to act unilaterally meant they could be highly pragmatic in their use of these associations. Where there was seen to be an advantage in acting collectively, they would look to advance their interests through this channel. However, where there appeared to be an advantage to the company in acting independently, they were prepared to do so. As the representative of a national trade association explained:

"And sometimes it’s also quite powerful for companies to take a different position to the rest of the sector because they can say to government and people, ‘Look, we’re the responsible guys here. It’s those others you’ve got a problem with’ in lots of areas that they’d be dealing with them. So sometimes our members like to have a different position to everybody else, to say that, ‘We’re the ones who are differentiating and we’re different. So we’re up here and they're down there somewhere’."

Breaking ranks with their competitors allows the company in question to stay ahead of debates on issues such as minimum pricing and to present themselves as industry leaders. Rather than being part of the problem of alcohol related harm, they can present themselves as an integral part of the solution, at the forefront of new measures and initiatives to tackle the issue.

Policy makers may also find it useful to exploit the cleavages between certain actors in order to drive forward their policy agenda. As a former Minister of Public Health put it:

"So I mean the trade associations were helpful, but do you know sometimes just trying to find, I think in this area, trying to find a leader in that sector who’s prepared to break from the pack and do something different, or be prepared to think about things differently, is actually a really… I think that’s a really good win if you can do that."

This point was confirmed by the representative of an industry trade association:

"But often officials will pick and choose and they’ll play people off against one another so I’ll sit in a meeting, they’ll say, ‘That’s not what one of your members told me last week.’ And so they’ll pick and choose stuff to try and cajole people on. They’ll often use one company against another company as well, to say, ‘Well, if they can do it, why can’t you do it?’ Those sorts of things."

Thus, whilst actors are keen to highlight the advantages of collective action, there are also potential benefits to be gained from presenting an individual viewpoint on certain issues. This is especially the case where an industry actor is able to fall in line behind a particular government initiative. Recognising the direction of travel of the policy debate and staying ahead of the trend enables companies to position themselves as responsible actors and as partners in the policy process.

In addition to trade associations it is necessary to highlight the role played by ‘social aspects’ organizations such as the Portman Group (PG). The PG was formed by, and continues to be governed and funded by, the UK’s leading alcohol producers. Initially founded to speak on behalf of its membership on the social aspects of alcohol, its role in promoting responsible drinking has since been handed over to Drinkaware. The PG’s main remit is now the implementation of its voluntary code of practice on the promotion and packaging of alcoholic beverages. However, it continues to represent the perspectives of producers on alcohol related issues both to politicians and through the media.

Whilst representatives from the PG and its member companies are keen to highlight that it is not a trade association and argue that it does not seek to further industry objectives, commentators from outside the industry see it as a mouthpiece for the industry [22]. A representative from Alcohol Focus Scotland, for example, recalled that it was often the PG – along with trade associations such as the WSTA – who would represent the industry perspective in the public debates on MUP in which she participated. Similarly, the PG felt it appropriate to make a submission to the Scottish Government’s consultation on the Alcohol Etc (Scotland) Bill. The appearance of autonomy from the industry is important to the function of the PG. It gives the organization a credibility and status not afforded to trade associations whose remit is more overtly to represent the interests of its members. Consequently, the PG has been afforded an important role not just in the regulation of alcohol marketing, but was given great prominence within previous governments’ alcohol strategies [23].

### Connections and co-operation within the alcohol industry

The existence of trade associations and the reliance on them by many organisations is indicative of the perceived importance of coordination by industry actors, despite their sometimes conflicting interests. There was a widespread belief amongst respondents that the ability of the industry (or particular sectors of it) to speak to government with a single voice was extremely beneficial in influencing the direction of policy in areas in which corporate actors have shared interests. Consequently, there is a certain paradox at the heart of the relationship between alcohol industry actors. On the one hand they are rival companies competing for a share of their respective markets, whilst on the other they often share strategic interests which they are more effectively able to defend when acting collectively. Gerard Hastings from the Stirling Management School argues that the situation faced by the alcohol industry is similar to that of the tobacco industry:

"The comparison with tobacco is good; once you get to producer level… the different tobacco companies hate each other, but, you know, on occasion, they will come together and try and work together."

It was evident from the interviews conducted that there are frequent contacts between industry actors through a variety of forums. Most obviously, representatives of different organisations meet within the context of trade associations. Given that many organisations are members of multiple trade associations, the contacts between their representatives are frequent. This can lead to the formation of strong and enduring personal relationships between the individual representatives of different organisations within these forums. As the representative of a national chain of supermarkets commented:

"I mean I’ve been a lobbyist for a good number of years, the lobbying community in Westminster is relatively small – I mean everybody knows everybody else; everybody has worked with everybody else – and then you go into the food retail lobbying group, you know, we all know each other, we see each other at least once or twice a week, we go to the same meetings, so everybody knows each other. I mean I’ve… the Head of Corporate Affairs from [another supermarket chain] used to be my boss in another job, so everybody knows each other terribly well."

Given their shared objectives, this respondent continued, the public affairs departments of many corporations often have more of an affinity with their equivalents in other companies than with different departments within their own organisations:

"I mean, you know, in some ways I have more in common with the Head of Public Affairs at [another supermarket chain] than I do with the buyers in [my company], because we kind of think in the same way, and we share the same frustrations about our businesses and the way in which they react; so often, you know, I’ll go in and complain about the way in which the issue’s being handled, and they’ll have the same kind of frustrations; […] I might say ‘oh I’m finding it really difficult to get the business to agree which of the definitions of below-cost is best for the business, because they don’t actually understand what the differences are.’ And then, you know, you’ll find that exactly the same thing’s happening in another business."

There was evidence also that individual corporations are prepared to co-ordinate their approach to government in certain circumstances. One such example is the coordinated approach between two companies with regard to one of the All Party Parliamentary Groups. Given the presence both companies have in the constituency of the Group Chairman, they were keen to be involved in it. Asked if he had been involved personally with the group, a representative of one of the companies commented:

"The short answer is I have not; that has been most of the trade associations, however because there’s now a constituency interest with [the Group Chairman], I want to be involved because I want to be able to be of service to him in terms of that; and [the other company] and I have talked about this, and we’re, you know, we’re both aligned in terms of that’s how we want to handle it with him just to make sure that we’re covered … there and to work with the trade associations in terms of communicating and whatnot."

Asked to clarify whether it was normal for companies to coordinate their approaches in this way, the respondent explained that there are extensive personal connections between individuals within different organisations:

"I mean normally ‘no’ is the short answer, but … in terms of working with [the Group Chairman], that’s where we have that conversation. But in all the trade bodies I’m sitting next to somebody… or the trade bodies or the Portman Group or something like that, constantly sitting next to somebody not only from [the other company], but also [people from other companies]; same thing in the trade associations – we all know each other, because in addition to that we have issues between the various companies…"

In addition to the contacts between corporations there are also extensive links between different trade associations. The representatives of one producer organisation commented, for example, that they are in contact with other trade associations ‘on a daily basis’. Similarly, representatives of the ALMR are also in contact with the BBPA and the British Institute of Inn Keeping, which also represent the on-trade. There are close relationships between the BRC and the WSTA, the main trade associations representing the off-trade sector. In addition to those companies which are members of both associations, the WSTA is itself a member of the BRC. The chief executive officers of four of the main alcohol trade associations meet together quarterly in a group called Trade Association Directors (TAD). The lines of communication between associations thus include both formalised channels of communication between the senior executives of the major trade associations and more informal, *ad hoc* discussions.

Alongside both the regular and *ad hoc* dialogue between associations, there is evidence of more strategic coordination between trade associations in different areas. This manifests itself in a division of labour between trade associations, whereby those with a particular interest in certain issues, or a specific expertise or geographical focus, will take the lead on a given issue. The clearest example of industry coordination of this type occurred in Scotland where the Scotch Whisky Association (SWA) took the lead on the issue of MUP, reflecting their specific knowledge of Scottish politics, the economic importance of the whisky industry in Scotland and the emblematic nature of whisky for Scottish identity [21].

## Discussion and conclusions

The alcohol industry consists of some of the largest corporations in the UK, as well as a number of smaller companies. Given the structure of the industry and the varying effects minimum pricing would have on different sectors, it is unsurprising that cleavages should appear. This article examines divisions between various industry actors and assesses the impact on their ability to organise collectively to influence policy. Our analysis indicates that industry actors were highly divided on the issue of MUP and related questions. Nevertheless, a number of coalitions emerged between different sectors which reflected shared interests or longstanding historical ties. Whilst there were often sharp disagreements between actors on substantive issues, there was a general agreement that collaboration between actors offered distinct advantages to all. Presenting a clear, unified message was ultimately seen as the most effective way to engage in policy debates. The dilemma faced by industry actors then was how to manage the conflict between such coordination and the need to advance their specific corporate or sectoral interests.

Many of the cleavages between actors are rooted in clear material interests which manifest themselves in the positions adopted on MUP, bans on below costs selling, or the levels of taxation levied on particular product categories. Whilst there was much disagreement between actors on substantive issues, there was widespread agreement about the desirability of the industry speaking with a single voice in its dealings with government. Trade associations are seen as an important channel for engaging with government because they are able to speak on behalf of the entire industry or, at least, a particular sector. However, it may be difficult for associations to arrive at a common position on the most controversial issues and so their effectiveness may be diluted by their need to accommodate all view points. In addition, the largest and politically most influential corporations can be highly pragmatic in their use of associations, seeking backing for their position amongst the membership of the association where possible, and breaking ranks when there is an advantage in so doing. Alongside trade associations, the interests of many of the largest producer companies are represented by the Portman Group. Despite its claims to autonomy, the group maintains close links with the member companies which fund it and is afforded a prominent place by government in policy debates [22].

Our study was limited in that some of those approached for interview declined or did not respond (see Table 1). Furthermore, it depended primarily on what interview respondents were willing to share with us and on information that was present in publically available documents. The increasing public perception of a need to find solutions to alcohol related problems has placed pressure on the industry, which may explain why some respondents appeared to lay the blame for such problems with other sections of the industry. Claims of industry fragmentation in interview responses may be in the interests of certain actors, who can thus present themselves as doing all they can in difficult circumstances. However, it may also be contrary to the interests of the industry as a whole, since it implies that only government regulation can be successful in imposing solutions on such a fragmented industry. This realisation is partially reflected in the continued attempts to agree common industry positions. We were able to mitigate these inherent problems of interpretation by triangulating data from respondents from all sectors of the industry and with those of other stakeholders with intimate knowledge of the alcohol policy process.

Alcohol industry corporations and trade associations collaborate with one another effectively where there are shared interests, allowing the best placed bodies to lead on a given issue. Thus, whilst industry actors may be deeply divided on certain issues they are able to coordinate their positions on occasions where there are clear advantages in so doing. Alongside more formal arrangements, this appears to be facilitated by the personal relationships and shared perspectives of industry personnel, particularly from companies’ public affairs departments.

The internal divisions of the industry appear to offer some hope for public health actors attempting to influence alcohol policy. As recent moves to implement MUP in Scotland and England demonstrate, access to policy makers does not necessarily translate into influence. The Scottish debates in particular highlight what can be achieved by a well organised and unified public health lobby. The ability of public health actors to articulate a clear and consistent policy position played a key role in bringing MUP onto the agenda, even in the face of strong industry opposition [21]. Nevertheless, industry actors remain extremely influential. They are highly pragmatic and well resourced actors. Larger actors can engage government effectively when acting alone as well as in unison. The long term relationships they cultivate with decision makers ensure they are seen as key stakeholders and retain a seat at the policy-making table, despite the divisions which may exist. Whilst industry actors have the resources and channels of influence to act effectively even where divisions exist, this is not the case for public health actors. The debates on MUP demonstrate the vital importance of coherence and coordination for public health advocates when engaging in policy debates [21]. Policymakers may also benefit from an awareness of the multiplicity of interests within the industry and the ways that these may shape collective positions, such as those articulated by trade associations, when formulating and consulting on policy.

Although the diverse range of actors comprising the industry is well established [15,16], this is the first study to examine how internal industry dynamics affect their ability to organise politically. As such, it provides a foundation for further research into the processes by which industry actors attempt to influence alcohol policy.

## Abbreviations

ACS, Association of Convenience Stores; ALMR, Association of Licensed Multiple Retailers; BBPA, British Beer and Pub Association; BRC, British Retail Consortium; GVA, Gin and Vodka Association; MUP, Minimum unit pricing; NACM, National Association of Cider Makers; PG, Portman Group; SWA, Scotch Whisky Association; WSTA, Wine and Spirits Trade Association.

## Competing interests

The authors declare that they have no competing interests.

## Authors’ contributions

CH led study conception, design, and securing funding. He contributed to the analysis and helped to draft the manuscript. BH conducted the interviews, analysed and coded the data. He produced the first draft of the manuscript and contributed to subsequent versions. JM contributed to design, securing funding, analysis and helped to draft the manuscript. All authors read and approved the final manuscript.

## Pre-publication history

The pre-publication history for this paper can be accessed here:

http://www.biomedcentral.com/1471-2458/12/483/prepub
